# Clinical and laboratory predictors of testicular loss in pediatric torsion patients

**DOI:** 10.1080/20905998.2026.2625609

**Published:** 2026-02-03

**Authors:** Zlatan Zvizdic, Una Glamoclija, Asmir Jonuzi, Nadja Zvizdic, Semir Vranic

**Affiliations:** a Department of Pediatric Surgery, Clinical Center University of Sarajevo, Sarajevo, Bosnia and Herzegovina; b Scientific Research Unit, Bosnalijek d.d., Sarajevo, Bosnia and Herzegovina; c Department of Biochemistry and Clinical Analysis, Faculty of Pharmacy, University of Sarajevo, Sarajevo, Bosnia and Herzegovina; d Department of Anesthesiology and Intensive Care, Garmisch-Partenkirchen Medical Center, Garmisch-Partenkirchen, Germany; e College of Medicine, QU Health, Qatar University, Doha, Qatar

**Keywords:** Testicular torsion, testicular salvage, symptom duration, clinical and laboratory parameters, pediatric patients

## Abstract

**Objectives:**

Pediatric testicular torsion (TT) requires emergency surgical intervention to prevent testicular loss. This study aimed to examine clinical and laboratory parameters that predict intraoperative orchidectomy and testicular atrophy (TA) at one-year follow-up, with a focus on symptom duration and inflammatory biomarkers.

**Materials and methods:**

A retrospective cohort study included 90 pediatric patients ( < 18 years) who underwent surgical exploration for TT. Clinical predictors included age, symptom duration, scrotal findings (pain, swelling, redness, high-riding testis), and preoperative preparation time. Laboratory parameters included leukocyte count, platelet count, mean platelet volume (MPV), and C-reactive protein (CRP). Testicular vascularization (normal vs. pathologic) was assessed preoperatively at presentation using color Doppler ultrasound (CDUS). Outcomes were orchiectomy or orchiopexy, and testicular atrophy (≥80% volume loss) at one-year follow-up. Multivariate logistic regression was used to identify independent predictors of testicular loss.

**Results:**

Forty patients underwent orchidectomy, and 50 underwent orchidopexy. Among those treated with orchidopexy, TS at one-year follow-up was achieved in 36 patients (72%). Age and type of vascularization were significantly different between patients from the orchidectomy and orchidopexy groups. There was a notably larger number of patients experiencing scrotal redness, swelling, or hardness in the orchidectomy group in comparison to the orchidopexy group. CRP was significantly higher in the orchidectomy group (*p* = 0.002). Multiple logistic regression indicated considerably increased odds ratios of orchidectomy with symptom duration > 12 hours, younger age, longer preoperative preparation times, and elevated CRP.

**Conclusion:**

Delayed presentation ( > 12 hours), younger age, elevated CRP, and longer preoperative preparation time were identified as the strongest predictors of orchidectomy and subsequent testicular atrophy. Symptom duration remained the most critical determinant of failed testicular salvage. These findings highlight the importance of timely recognition and prompt surgical management to optimize outcomes in pediatric testicular torsion.

## Introduction

Testicular torsion (TT) is a urological emergency predominantly affecting pediatric and adolescent males, characterized by ischemia and potential testicular necrosis caused by twisting of the testis around the spermatic cord. Prompt diagnosis and treatment are critical for testicular salvage [1,2]. However, differentiating TT from other causes of acute scrotum can be challenging due to overlapping symptoms [3].

Recently, the Testicular Workup for Ischemia and Suspected Torsion (TWIST) score may potentially aid in differentiating TT from other causes of acute scrotum, incorporating five key clinical parameters: testicular swelling, hard testis, absent cremasteric reflex, nausea/vomiting, and high-riding testis [4]. Higher TWIST scores strongly correlate with TT and can guide urgent surgical decision-making, particularly in settings where imaging may be delayed. Although clinical symptoms and signs such as testicular pain, swelling, high-riding testis, absent cremasteric reflex, and decreased or absent blood flow on the ipsilateral side are key diagnostic indicators, inexpensive and widely available laboratory biomarkers, such as complete blood count and C-reactive protein (CRP) have recently been investigated for their potential role in assessing testicular viability (TV) and prognostic outcomes after orchidopexy, including the development of testicular atrophy (TA) during follow-up [5–8]. However, evidence remains limited regarding the prognostic value of these biomarkers in predicting the need for orchidectomy and long-term outcomes such as TA. Current predictors used to assess TV primarily include clinical presentation (severity and duration of pain, swelling, high-riding testis), physical examination findings such as absence of the cremasteric reflex, and color Doppler ultrasound (CDUS) assessment of intratesticular blood flow. Although these parameters remain fundamental in emergency decision-making, each has important limitations. Clinical findings may overlap with other causes of acute scrotum. Doppler ultrasound can yield false-negative results, particularly in early or intermittent torsion, and neither reliably predicts long-term outcomes such as testicular atrophy after orchiopexy. Laboratory biomarkers such as leukocyte count, NLR, and CRP have emerged as potential adjuncts, yet evidence remains inconsistent, and their prognostic accuracy remains uncertain [9–12]. These limitations highlight the need for more integrated, objective predictors capable of improving risk stratification and guiding timely surgical decision-making.

This study aimed to investigate clinical and laboratory parameters associated with intraoperative testicular loss and long-term prognostic outcomes after orchidopexy, with particular emphasis on symptom duration and preoperative inflammatory markers, to enhance risk stratification and optimize treatment approaches.

## Materials and methods

This retrospective cohort study included 90 pediatric patients < 18 years old who presented to the pediatric emergency department (ED) and underwent surgery for TT (International Classification of Diseases-10th Revision, ICD-10 code N44.00). The study was conducted at the Clinical Center, University of Sarajevo (Bosnia and Herzegovina), and included patients between January 2010 through December 2023. Patients were categorized into two groups based on surgical outcomes during scrotal exploration for TT: orchidectomy (*n* = 40) and orchidopexy (*n* = 50) groups (Figure 1). Furthermore, the orchidopexy group was subdivided into testicular salvage (TS) (*n* = 36) and TA groups (*n* = 14) (Figure 1).

**Figure 1.**
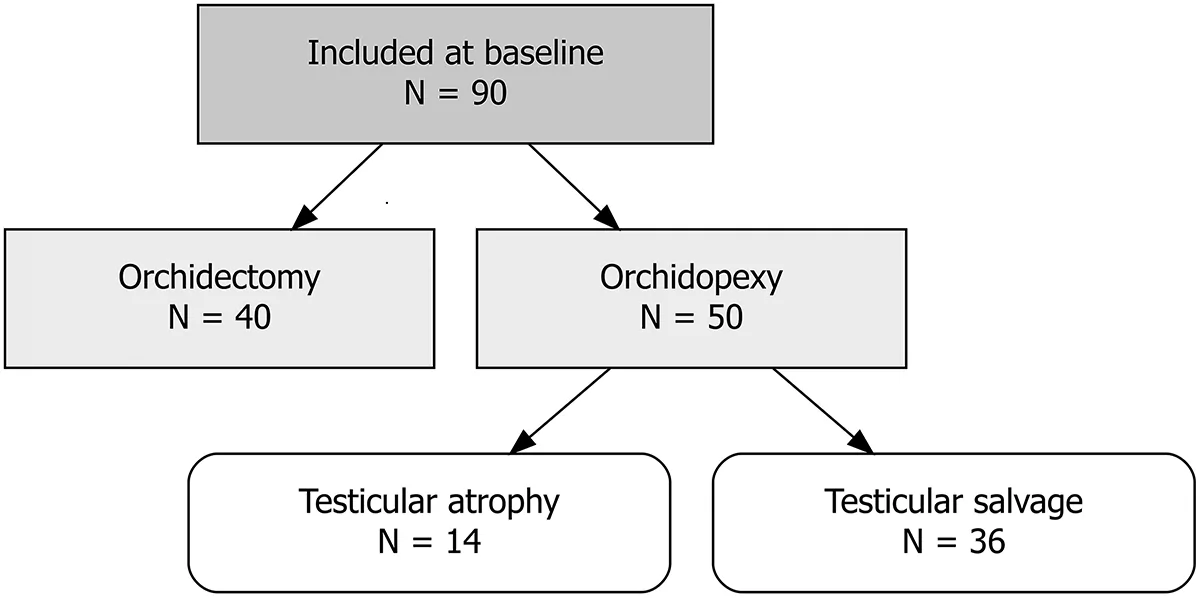
Flowchart of the study.

Patient data collected included age, laterality, symptoms, symptom duration, preoperative preparation time, preoperative complete blood count (CBC) (leukocyte count, mean platelet volume MPV, platelet count), preoperative CRP, preoperative CDUS findings, and outcome in the one-year follow-up period (TS or TA).

CRP and all CBC-derived parameters (leukocyte count, platelet count, hematocrit, and MPV) were routinely measured upon ED presentation as part of the standard preoperative laboratory panel for all patients with suspected TT.

All patients with surgically confirmed TT were included in the present study. Exclusion criteria were as follows: (1) Patients with a history of scrotal trauma; (2) Patients with perinatal (extravaginal) torsion diagnosed antenatally or at birth; (3) Absence of TT at surgical exploration, and (4) Refusal of orchidectomy by the parents or legal guardians. Importantly, neonates presenting with postnatal intravaginal testicular torsion were not considered perinatal TT and were therefore included in the analysis. Age-specific CBC reference ranges and MPV cutoff values were defined according to the Pediatric CBC Reference Values (LTR10211) [13]. An elevated MPV was defined as > 11.5 fl, while a decreased MPV was defined as < 7.5 fl, adjusting for the platelet count. Low platelet count was defined as presenting < 150 × 109/L, and elevated platelet count was defined as presenting > 450 × 109/L. Platelet values between these ranges were considered normal.

The duration of symptoms (DoS), defined as the time from the reported onset of symptoms to the patient’s presentation at the pediatric ED, was categorized into predefined intervals < 6 hours (very early), 6–12 hours, 12–24 hours, 24–36 hours, and > 36 hours to reflect clinically meaningful time windows (very early, early, intermediate, delayed, prolonged) [14,15] for descriptive statistics, and < 12 hours and > 12 hours in logistic regression modelling, since we had to collapse categories to ensure enough observations per group for stable estimates. Preoperative preparation time, or time to treat (TtT), was defined as the interval between the patient’s arrival at the pediatric ED and the start of the operation. DoS was retrieved from the standardized, time-stamped triage record documenting the caregiver-reported onset of pain at ED presentation. TtT was calculated using two objective electronic timestamps: the ED arrival time and the recorded operating room incision time. CDUS assessment of testicular blood flow was categorized as normal vascularization (preserved or symmetrical intratesticular blood flow compared with the contralateral testis) or as pathologic vascularization (decreased, absent, or heterogeneous flow consistent with ischemia secondary to torsion).

After identifying the type and degree of TT and repairing the twisted spermatic cord, the affected testis was warmed with sterile gauze soaked in warm saline. In all cases with equivocal viability after detorsion, a small incision in the tunica albuginea was performed using the three-grade bleeding test proposed by Arda and Ozyaylali [16] to assess bleeding and determine the need for orchidectomy. Histopathology was only done in orchidectomy cases as per standard practice. For all patients who underwent orchidopexy, postoperative outcomes were evaluated using standardized ultrasonographic follow-up at 3, 6, and 12 months, assessing testicular blood flow and calculating TV to determine TS or TA. TV was calculated using the formula 0.71 ×length ×width ×thickness of the testicular ellipsoid [17]. Testicular atrophy (TA) was defined as a postoperative testicular volume of less than 50% compared to that of the contralateral testis measured by ultrasonography, and severe TA as a testicular volume loss > 80% or the absence of sustained blood supply [18]. For outcome analyses, TA included both moderate (≥50%) and severe ( > 80%) volume loss.

The study was conducted following the principles of the Declaration of Helsinki, and a local institutional review board (Ethical Committee of the Clinical Center, University of Sarajevo) approved the study (approval 16–37/2023). The requirement for informed consent was routinely waived, as data were obtained from existing patient records, and de-identification of the records was applied to ensure the full confidentiality of personal information. All modifications and analyses were conducted programmatically, with a new database created; the original database remained unaltered.

## Statistical analysis

The shape of the data distribution was evaluated by visualization of box plots, histograms, and Q-Q plots. Continuous variables with normal distribution and no outliers were presented as mean ± standard deviation, and a Student t-test was applied for analysis if all assumptions were met. Continuous variables that did not follow a normal distribution or had outliers were presented as median (interquartile range, IQR), and the Mann-Whitney U test was used for analysis. Categorical variables were presented as numbers (percentage in total number), and Pearson’s Chi-squared test or Fisher’s Exact Test was used for analysis. The imputation of missing data was performed using the k-Nearest Neighbors method with distance-weighted mean in the VIM package in R. Multiple logistic regression was performed after analysis of missing values and appropriate data imputation. Two models were prepared: one for the binary outcome variable with orchidectomy as a comparison category and the other for the testicular salvage variable with no testicular salvage as a comparison category. The models included independent variables such as age, duration of symptoms, duration of preparation time, hematocrit, number of leukocytes and platelets, MPV, and CRP. The cross-validation of the models was performed using the caret package in R. R Statistical Software (Foundation for Statistical Computing, Vienna, Austria) version 4.4.2. was used with the following applied packages: readxl, tidyverse, ggpubr, gtsummary, labelled, ggplot2, naniar, rstatix, VIM, MissMech, car, pROC, and caret. *p* < 0.05 was considered statistically significant. All tests were two-sided.

## Results

The study cohort comprised 90 patients, with 40 undergoing orchidectomy and 50 undergoing orchidopexy (Figure 1). TS occurred in 36 patients (40% of the total cohort), corresponding to 72% of patients undergoing orchidopexy after one-year follow-up. DoS, age, and vascularization were significantly different between patients from the orchidectomy and orchidopexy groups. Although patients with perinatal TT were excluded, the orchidectomy group included a higher proportion of neonates with postnatal intravaginal torsion (seven neonates), compared with the orchidopexy group (one neonate), which contributed to the lower median age observed in the orchidectomy group (Table 1). Scrotal redness, swelling, and hardness were more frequently observed in patients who underwent orchidectomy, reflecting more advanced ischemic inflammatory changes. DoS before orchidopexy was significantly different between patients with and without TS at one-year follow-up (Table 1).

**Table 1.** Comparison of characteristics and clinical findings between patients who had orchidectomy or orchidopexy and patients with testicular atrophy and testicular salvage after one-year follow-up.

Characteristic	Overall, *N* = 90	Orchidectomy, *N* = 40	Orchidopexy, *N* = 50	p-value	Testicular atrophy *N* = 14	Testicular salvage *N* = 36	*p*-value
**Age, years†**	**13.6 (11.7, 15.4)**	**12.6 (0.7, 15.1)**	**14.2 (12.9, 15.5)**	**0.010**	14.7 (12.2, 15.8)	14.1 (12.9, 15.3)	0.487
Duration of symptoms, hours*				** <0.001**			** <0.001**
< 6	33 (36.7)	1 (2.5)	32 (64.0)		0 (0.0)	32 (88.9)	
6–12	8 (8.9)	1 (2.5)	7 (14.0)		5 (35.7)	2 (5.6)	
12–24	9 (10.0)	6 (15.0)	3 (6.0)		2 (14.3)	1 (2.8)	
24–36	9 (10.0)	7 (17.5)	2 (4.0)		1 (7.1)	1 (2.8)	
> 36	31 (34.4)	25 (62.5)	6 (12.0)		6 (42.9)	0 (0.0)	
Symptoms*							
Testicular pain	84 (93.3)	35 (87.5)	49 (98.0)	0.085	14 (100.0)	35 (97.2)	1.000
Scrotal swelling	57 (63.3)	**30 (75.0)**	**27 (54.0)**	**0.040**	9 (64.3)	18 (50.0)	0.363
Scrotal Redness/Darkening	36 (40.0)	**24 (60.0)**	**12 (24.0)**	**0.001**	4 (28.6)	8 (22.2)	0.718
Scrotal hardness	29 (32.2)	**19 (47.5)**	**10 (20.0)**	**0.006**	4 (28.6)	6 (16.7)	0.436
Normal appearance of the scrotum	3 (3.3)	2 (5.0)	1 (2.0)	0.583	1 (7.1)	0 (0.0)	0.280
Vomiting	20 (22.2)	8 (20.0)	12 (24.0)	0.650	3 (21.4)	9 (25.0)	1.000
Nausea	3 (3.3)	1 (2.5)	2 (4.0)	1.000	1 (7.1)	1 (2.8)	0.486
Febrility	2 (2.2)	2 (5.0)	0 (0.0)	0.195	0 (0.0)	0 (0.0)	1.000
Position*				0.662			0.242
High-riding testis	55 (61.1)	25 (62.5)	30 (60.0)		10 (71.4)	20 (55.6)	
Normal	28 (31.1)	10 (25.0)	18 (36.0)		3 (21.4)	15 (42.9)	
Unknown	7 (7.8)	5 (12.5)	2 (4.0)		1 (7.1)	1 (2.8)	
**Preparation time, minutes†**	80 (60, 105)	85.0 (60, 120)	80.0 (60, 90)	0.146	82.5 (75.0, 90.0)	70.0 (57.5, 95.0)	0.459
Testicle*				0.848			0.066
Right	37 (41.1)	16 (40.0)	21 (42.0)		3 (21.4)	18 (50.0)	
Left	53 (58.9)	24 (60.0)	29 (58.0)		11 (78.6)	18 (50.0)	
Vascularization*				** <0.001**			0.324
Normal	23 (25.6)	0 (0.0)	23 (46.0)		8 (57.1)	15 (41.7)	
Pathologic	67 (74.4)	40 (100.0)	27 (54.0)		6 (42.9)	21 (58.3)	

Data presented as *n (%) and analyzed using Pearson’s Chi-squared test or Fisher’s Exact Test, or †median (interquartile range, IQR) and analyzed using Mann-Whitney U test. Only statistically significant values are bolded. Neonates included in the analysis represent postnatal intravaginal torsion cases; patients with perinatal (extravaginal) torsion were excluded.

CRP values were significantly higher in patients from the orchidectomy group compared to the orchidopexy group (*p* = 0.002). Although platelet count was higher in the orchidectomy group (*p* = 0.021), no difference was seen regarding the proportion of patients with elevated platelets (*p* = 0.298) (Table 2). Platelet count was higher in the orchidectomy group (*p* = 0.021); however, elevated platelet levels did not differ significantly between TS and TA outcomes (*p* = 0.298) (Table 2).

**Table 2.** Laboratory findings in patients who had orchidectomy or orchidopexy and in patients with or no testicular atrophy after one-year follow-up.

Characteristic	Overall, *N* = 90	Orchidectomy, *N* = 40	Orchidopexy, *N* = 50	*p*-value	Testicular atrophy *N* = 14	testicular salvage *N* = 36	*p*-value
Leukocytes†, 10^9^/L	11.8 (8.8, 15.6)	12.2 (8.7, 16.5)	11.0 (8.8, 13.6)	0.130	12.5 (7.7, 13.6)	10.5 (9.2, 13.3)	0.795
Leukocyte categories*				0.107			0.476
High	26 (28.9)	15 (37.5)	11 (22.0)		4 (28.6)	7 (19.4)	
Normal	64 (71.1)	25 (62.5)	39 (78.0)		10 (71.4)	29 (80.6)	
Erythrocytes‡, 10^12^/L	5.0 (0.4)	4.9 (0.4)	5.1 (0.4)	0.067	5.2 (0.6)	5.0 (0.3)	0.173
Hemoglobin‡, g/L	145.7 (14.8)	144.9 (15.2)	146.3 (14.6)	0.665	149.1 (17.4)	145.2 (13.5)	0.304
Hematocrit†, %	42.0 (38.2, 45.6)	42.3 (38.7, 45.3)	41.9 (38.1, 46.0)	0.592	42.0 (38.1, 45.0)	41.4 (38.1, 45.0)	0.482
MCV†, fL	83.0 (79.0, 86.8)	83.5 (79.7, 86.9)	82.5 (79.0, 86.0)	0.253	80.4 (78.0, 85.0)	83.0 (79.0, 86.4)	0.309
MPV†, fL	7.1 (6.8, 7.6)	7.0 (6.8, 7.5)	7.2 (6.8, 7.8)	0.131	7.5 (6.7, 7.9)	7.2 (6.9, 7.7)	0.914
MPV categories*				0.134			0.368
Low	60 (66.7)	30 (75.0)	30 (60.0)		7 (50.0)	23 (63.9)	
Normal	30 (33.3)	10 (25.0)	20 (40.0)		7 (50.0)	13 (36.1)	
**Platelets**†, **10**^**9**^**/L**	**276 (238, 318)**	**283 (253, 337)**	**266 (233, 291)**	**0.021**	277 (230, 310)	260 (234, 288)	0.358
Platelet categories*				0.298			0.126
High	12 (13.3)	7 (17.5)	5 (10.0)		3 (21.4)	2 (5.6)	
Normal	78 (86.7)	33 (82.5)	45 (90.0)		11 (78.6)	34 (94.4)	
**CRP**†, **mg/L**	**0.6 (0.4, 10.2)**	**6.3 (0.4, 29.9)**	**0.4 (0.4, 5.5)**	**0.002**	0.7 (0.4, 22.6)	0.4 (0.4, 5.1)	0.124
CRP categories*				**0.023**			0.704
**High**	**30 (33.3)**	**17 (42.5)**	**13 (26.0)**		4 (28.6)	9 (25.0)	
**Normal**	**46 (51.1)**	**14 (35.0)**	**32 (64.0)**		7 (50.0)	25 (69.4)	
**Unknown**	**14 (15.6)**	**9 (22.5)**	**5 (10.0)**		3 (21.4)	2 (5.6)	

Data presented as: *n (%) and Pearson’s Chi-squared test or Fisher’s Exact Test were used for analysis, †median (interquartile range, IQR) and Mann-Whitney U test were used for analysis, or ‡mean (± standard deviation) and Student t-test were used for analysis.

MCV = Mean Corpuscular Volume; MPV = Mean Platelet Volume; CRP = C-reactive protein.

Only statistically significant values are bolded.

Multiple logistic regression was performed with the outcome variable, and orchidectomy was used as a comparison category. The model included independent variables: age in years, duration of symptoms with categories < 12 hours and > 12 hours, duration of preparation time in minutes, hematocrit, leukocyte and platelet counts, MPV, and CRP. The model had an Akaike Information Criterion (AIC) value of 90.075, McFadden’s R-squared value of 0.417, and an area under the curve (AUC) value of 0.887, indicating a strong fit. Model cross-validation showed an accuracy of 0.800 and Cohen’s Kappa of 0.588. The odds ratio of orchidectomy was significantly increased with DoS for more than 12 hours, longer preparation times, and higher CRP. We observed a decreased odds ratio of orchidectomy with advanced age (Table 3). A similar model was prepared for the testicular salvage variable (TS included orchidopexy patients who had TS after one year of follow-up, and no TS included all other patients). The only positive predictor of no testicular salvage was duration of symptoms for more than 12 hours, with an odds ratio of 120 (95% CI 19–1445). The model had an AIC value of 69.421, McFadden’s R-squared value of 0.576, and an AUC value of 0.935, indicating a strong fit. Model cross-validation showed an accuracy of 0.883 and Cohen’s Kappa of 0.760.

**Table 3.** Odds ratios (OR) with 95% confidence intervals (CI) for orchidectomy in a model of multiple logistic regression.

Independent variable	OR (95% CI)	p-value
Age, years	**0.87 (0.76, 0.98)**	**0.027**
Duration of symptoms, > 12 hours	**11.1 (3.01, 50.4)**	**0.001**
Preparation time, minutes	**1.02 (1.00, 1.03)**	**0.024**
Hematocrit, %	0.93 (0.81, 1.07)	0.332
Leukocytes, 10^9^/L	1.06 (0.90, 1.25)	0.514
Platelets, 10^9^/L	1.00 (0.98, 1.01)	0.402
MPV, fL	0.63 (0.30, 1.23)	0.190
CRP, mg/L	**1.06 (1.02, 1.12)**	**0.019**

CRP: C-reactive protein, MPV: Mean Platelet Volume. Only statistically significant values are bolded.

## Discussion

This study comprehensively evaluated clinical presentation, laboratory biomarkers, procedural timing, ultrasonography, and multivariate risk factors to elucidate predictors of orchidectomy and long-term TA in pediatric TT. This retrospective study highlights the critical importance of timely diagnosis and surgical intervention in pediatric TT. Our findings show that DoS, CRP levels, and preoperative preparation time significantly influence surgical outcomes, particularly the likelihood of orchidectomy and long-term TS. These results are consistent with previous studies indicating that TV is time-sensitive and declines rapidly beyond 6–12 hours of ischemia [1,5,19,20].

Our finding that DoS > 12 hours significantly predicts TS failure reflects contemporary data suggesting an optimal DoS of ≤9 hours for orchidopexy success and TS. This is further supported by a multicenter Chinese study that identified DoS as the strongest predictor of orchiectomy (OR 1.11 per hour of delay) [7].

We observed that elevated CRP independently predicted orchidectomy. Although our results showed platelet count differences between groups, they did not reach independent predictive value, unlike CRP. Elevated CRP was strongly associated with orchidectomy, likely reflecting more advanced ischemic injury rather than exerting a causal effect. Although CRP likely reflects delayed ischemic injury rather than a causal mechanism, it retained independent predictive value after adjustment for symptom duration. Meanwhile, MPV’s role remained inconclusive. A recent meta-analysis did not find a significant association between CRP and salvage, but it linked higher MPV with an increased risk of orchiectomy [21]. Similarly, He et al. found that MPV remained an independent predictor of orchiectomy (OR 3.7; cut-off 10.55 fL) [7]. Differences between our results and previous studies need to be verified in prospective studies with larger sample sizes.

Our study identified increased WBC and platelet counts in the orchidectomy and TA groups; nevertheless, these findings lost significance in the multivariate analysis. The existing literature indicates contradictory results: some studies associate higher neutrophil-to-lymphocyte ratio (NLR), platelet-to-lymphocyte ratio (PLR), and WBC with the diagnosis and prognosis of TT [22], while other studies indicate that PLR or MPV have limited usefulness in predicting outcomes [23].

Younger age was independently associated with an increased likelihood of orchidectomy. This finding should not be attributed to the inclusion of perinatal TT, as patients with perinatal (extravaginal) TT were explicitly excluded from the study. Instead, this association is explained by the inclusion of neonates and very young infants with postnatal intravaginal torsion, a subgroup known to present with delayed or atypical symptoms and to have a lower testicular salvage rate. Diagnostic delay in this age group is well recognized and likely contributes to the increased risk of testicular loss [24]. Clinically, scrotal redness, swelling, and hardness occurred notably more often in cases requiring orchidectomy, underscoring the need for urgent evaluation [25]. These clinical signs likely represent advanced local inflammation secondary to prolonged ischemia, which helps explain their higher prevalence among patients requiring orchidectomy. Our logistic regression models demonstrated robust performance (AUCs of 0.89 for orchidectomy and 0.94 for TS). This model confirms that longer DoS, younger age, extended preparation time, and elevated CRP are the most robust predictors of orchidectomy. No additional predictive value was observed for other hematologic markers after accounting for these factors. These models, incorporating objective predictors such as CRP, DoS, and timing of surgery, provide a solid foundation for decision-support tools in emergency settings. Prompt surgical readiness protocols could potentially reduce orchidectomy rates.

Limitations of the present study include its retrospective design and potential selection and recall biases, particularly regarding symptom onset. A single-center design also limits external validity. Furthermore, variability in MPV measurement, influenced by age and laboratory methodology, suggests caution. In interpreting postoperative TA, we acknowledge that comparing the affected testis with the contralateral side may introduce minor inaccuracy due to natural inter-testicular size variability. However, this method remains the most widely accepted and practical reference standard in pediatric TT research, particularly in retrospective designs, and therefore was used for consistency with established literature. A prospective, multicenter validation of our models is warranted, preferably incorporating serial assessments of inflammatory markers to enhance their diagnostic and prognostic value.

Our study emphasizes the importance of prompt diagnosis and surgical intervention in pediatric TT. Delays exceeding 12–36 hours, elevated CRP, and prolonged pre-operative delays can greatly threaten testicular viability. Although MPV shows promise, its role in predicting intraoperative testicular viability or TS in the one-year follow-up period remains uncertain. Future efforts should focus on integrating key clinical and laboratory variables into emergency triage algorithms to increase the definitive TS rate.

## Data access statement

Available from the corresponding author upon reasonable request.

## Abbreviations

AIC: Akaike Information Criterion
AUC: Area under the curve
CDUS: Color Doppler ultrasound
CI: Confidence interval
CRP: C-reactive protein
DoS: Duration of symptoms
ED: Emergency department
IQR: Interquartile range
MPV: Mean platelet volume
NLR: Neutrophil-lymphocyte ratio
PLR: Platelet-lymphocyte ratio
TT: Testicular torsion
TtT: Time to treat
TS: Testicular salvage
CBC: Complete blood count
TV: Testicular viability
